# The role of resting myocardial blood flow and myocardial blood flow reserve as a predictor of major adverse cardiovascular outcomes

**DOI:** 10.1371/journal.pone.0228931

**Published:** 2020-02-13

**Authors:** Marie A. Guerraty, H. Shanker Rao, Venkatesh Y. Anjan, Hannah Szapary, David A. Mankoff, Daniel A. Pryma, Daniel J. Rader, Jacob G. Dubroff

**Affiliations:** 1 Division of Cardiovascular Medicine, Department of Medicine, University of Pennsylvania, Philadelphia, Pennsylvania, United States of America; 2 Department of Genetics, University of Pennsylvania, Philadelphia, Pennsylvania, United States of America; 3 Prairie Cardiovascular, Belleville, Illinois, United States of America; 4 Division of Nuclear Medicine and Molecular Imaging, Department of Radiology, University of Pennsylvania, Philadelphia, Pennsylvania, United States of America; 5 Division of Translational Medicine, Department of Medicine, University of Pennsylvania, Philadelphia, Pennsylvania, United States of America; Southeast University, CHINA

## Abstract

Cardiac perfusion PET is increasingly used to assess ischemia and cardiovascular risk and can also provide quantitative myocardial blood flow (MBF) and flow reserve (MBFR) values. These have been shown to be prognostic biomarkers of adverse outcomes, yet MBF and MBFR quantification remains underutilized in clinical settings. We compare MBFR to traditional cardiovascular risk factors in a large and diverse clinical population (60% African-American, 35.3% Caucasian) to rank its relative contribution to cardiovascular outcomes. Major adverse cardiovascular events (MACE), including unstable angina, non-ST and ST-elevation myocardial infarction, stroke, and death, were assessed for consecutive patients who underwent rest-dipyridamole stress 82Rb PET cardiac imaging from 2012–2015 at the Hospital of the University of Pennsylvania (n = 1283, mean follow-up 2.3 years). Resting MBF (1.1 ± 0.4 ml/min/g) was associated with adverse cardiovascular outcomes. MBFR (2.1 ± 0.8) was independently and inversely associated with MACE. Furthermore, MBFR was more strongly associated with MACE than both traditional cardiovascular risk factors and the presence of perfusion defects in regression analysis. Decision tree analysis identified MBFR as superior to established cardiovascular risk factors in predicting outcomes. Incorporating resting MBF and MBFR in CAD assessment may improve clinical decision making.

## Introduction

There is growing interest in coronary microvascular disease (CMVD) which has been associated with worsened cardiovascular outcomes [1] and may be involved in the pathogenesis of heart failure [2, 3]. PET (positron emission tomography) has been used extensively for myocardial perfusion imaging (MPI) given superior imaging characteristics compared to SPECT (single photon emission computed tomography) [4]. In addition to enhanced MPI, cardiac PET can provide quantitative myocardial blood flow (MBF) and flow reserve (MBFR) values, defined as the ratio of hyperemic MBF to resting MBF [5]. In the absence of epicardial coronary artery disease, MBFR is a measure of coronary microvascular function. Low MBFR has been shown to correlate with worsened outcomes in various populations, including patients with diabetes, renal disease, and obesity and patients without coronary artery disease (CAD) [6–10]. With the availability of several commercial software packages to quantify MBF and MBFR, PET has reproducibly shown value as a prognostic biomarker of adverse outcomes [11–15].

Yet MBFR quantification remains clinically underutilized. Likely reasons for this include limited availability of imaging modalities that allow the quantification of MBFR; limited experience in understanding the pathophysiology and interpretation of decreased MBFR; nascent understanding of the risk factors that contribute to MBFR; and unclear understanding of the role of MBFR as an independent cardiovascular risk factor. In a general population, the interpretation of MBFR is often confounded by epicardial coronary artery disease, which can affect regional radioactive tracer uptake, and therefore, regional blood flow calculations [16].

To provide clinical context and understand the relationship between established cardiovascular risk factors and MBFR in a large and diverse clinical population, we examined consecutive patients referred for cardiac Rubidium-82 (82Rb) PET at the Hospital of the University of Pennsylvania, a tertiary referral center, over a 2.3 year period for risk factors and cardiovascular outcomes. We hypothesized that MBFR has superior predictive value for cardiovascular events beyond that provided by traditional cardiovascular risk factors (such as hypertension and diabetes) or perfusion defects. We performed regression analysis and unbiased decision-tree and binary-discretization analyses to understand the relationships between cardiovascular risk factors, MBFR, and cardiovascular outcomes and rank the relative contributions of MBFR and cardiovascular risk factors to outcomes.

## Materials and methods

All patients undergoing clinical cardiac PET 82Rb myocardial perfusion imaging at the Hospital of the University of Pennsylvania, an urban tertiary care center, between 2/2012 and 4/2015 were retrospectively examined in the study. Patients were included in the regression and outcomes analyses if they had both resting and stress MBF measurements. Heart transplant patients represent a unique population and were excluded from adjusted and survival analyses. The patient records were followed through 1/2016 with mean follow-up of 2.3 years. Cardiovascular outcomes were assessed blinded to MBFR and by manual chart review of the University of Pennsylvania Health Systems Electronic Health Record. They include unstable angina, non-ST elevation myocardial infarction, ST-elevation myocardial infarction, stroke, or death, and were identified in one of three ways: described in a physician visit note or discharge summary, identified by brain and coronary imaging for acute stroke and MI, respectively; or assessed by ICD-9 code following the time of PET scan. The study was approved by the University of Pennsylvania Institutional Review Board, and no informed consent was required for this retrospective study using data from the Electronic Health Record.

Patients underwent a rest-dipyridamole stress 82Rb cardiac PET using Siemens Biograph mCT PET/CT scanner. Briefly, low dose CT images were acquired for photon attenuation correction. Rest images were obtained with a 6-minute list-mode dynamic PET acquisition imaging while 30mCi of 82Rb were injected intravenously as a fast bolus. Dipyridamole (0.56 mg/kg) was then administered, and three minutes after completion of dipyridamole infusion, dynamic PET imaging was repeated with an additional 30 mCi of 82Rb. Iterative reconstruction was performed with 2 iterations and matrix size 128 x 128.

Global and regional MBF and MBFR were calculated using syngo^®^ MBF software (Siemens Healthcare, Germany). The software uses the data from list-mode acquisition to determine time-activity curves for blood pool and myocardium. The data are fit into a one-compartment model of 82Rb kinetics with non-linear extraction curve to calculate global and regional MBF [17]. This methodology including variable extraction fraction has been validated and adjusted using microsphere studies including direct comparison with 82Rb in a porcine model [17–19]. The syngo^®^ platform calculates the blood input function by identifying maximum activity points in the late summed image and performing subsequent LV motion correction. Assuming an intra-ventricular cylindrical-spherical shape model, tissue uptake time activity curves are generated from the maximum activity points obtained. There is minimal inter-and intra-observer variability in using the software [19].

Absolute numbers and percentages are used to describe the patient population. Continuous variables are expressed as mean +/- standard deviation and were assessed for normality using Kolmogorov-Smirnov test [20, 21]. Variables that were not normally distributed were log-transformed. Student’s T-test was used to compare groups, and P-values<0.05 were considered significant. Adjusted and unadjusted regression modeling and Kaplan Meier survival analysis were performed using R version 3.2.3 [22, 23]. Adjusting MBF by heart rate-blood pressure (HRBP) product did not alter the results, and therefore the unadjusted MBF values are presented. Discretization analysis was performed using entropy-based approach [24]. Decision tree analysis was performed using weka-3-13. Risk factors examined included age, gender, race, BMI, global rest MBF, global MBFR, the presence of perfusion defects, diabetes, hypercholesterolemia, family history of cardiac disease, obstructive sleep apnea, presence of hypertension, CKD, renal transplant, and tobacco use [25].

## Results

Patient demographics are summarized in Table 1 and illustrate the age (58 ± 12.1), gender (54.9% female), and racial diversity (60% African-American) of the population. The population reflects institutional referral practices where PET myocardial imaging perfusion is preferred to SPECT imaging for high BMI and high-risk patients in our center. This referral bias leads to a population with high BMI (36.4 ± 10.0), a high incidence of cardiovascular risk factors, and many patients who had undergone renal or heart transplant. Additionally, a significant proportion had a history of CAD (41.1%) by ICD-9 diagnosis code. Given the higher rate of medical comorbidities, the rate of catheterization was 25% and the rate of cardiovascular events over the median follow-up of 27.6 months was 5%.

**Table 1:** Patient characteristics.

	n (%) or mean +/- SD
Age, y	58 ± 12.1
Gender	
Male	579 (45.1)
Female	704 (54.9)
Race	
African-American	768 (60)
Caucasian	453 (35.3)
Asian	23 (1.8)
Hispanic	19 (1.5)
Other/unknown	20 (1.6)
Body Mass Index	36.4 ± 10.0
Hypertension	1065 (83)
Diabetes Mellitus	581 (45.3)
Hypercholesterolemia	820 (64.0)
Coronary Artery Disease	528 (41.1)
Congestive Heart Failure	362 (28.2)
Stroke	115 (9.0)
Peripheral Artery Disease	82 (6.4)
Chronic Kidney Disease	445 (34.7)
Family History of Heart Disease	101 (7.9)
Renal Transplant	178 (13.9)
Heart Transplant	202 (15.7)
Tobacco Use	
Never	478 (37.2)
Former	496 (38.7)
Current	149 (11.6)
Other/Unknown	160 (12.5)
Indications	
Chest pain	684 (53.3)
Pre-kidney transplant	112 (8.7)
Pre-operative assessment	121 (9.4)
Post-heart transplant	69 (5.4)
Cardiomyopathy or CHF	33 (2.6)
To evaluate known CAD	35 (2.7)
Arrythmia	23 (1.8)
Syncope/Dizziness	21 (1.6)
Abnormal EKG	12 (1.0)
Other/Unknown	169 (13.2)
Laboratory Values	
Hemoglobin (g/dL)	12.7 ± 2
Hemoglobin A1c (%)	6.8 ± 1.8
Glucose (mg/dL)	125.5 ± 40.3
Creatinine (mg/dL)	1.6 ±1.8
Estimated GFR (ml/min/1.73m2)	33.9 ± 18.5
Pro-B Natriuretic Peptide (ng/L)	1392.1 ± 3095.9
Total Cholesterol (mg/dL)	168.8 ± 43.7
LDL Cholesterol (mg/dL)	95.7 ± 45.3
HDL Cholesterol (mg/dL)	46.3 ± 15.3
Non-HDL Cholesterol (mg/dL)	120.9 ±40.7
Triglycerides (mg/dL)	139.5 ± 90.6
Radioisotope Dose	
Rest Dose (mCi)	27.8 ± 1.6
Stress Dose (mCi)	27.7 ± 2.1
Perfusion	
No defect	996 (78.0)
Fixed or reversible defect	281 (22.0)
Global Myocardial Blood Flow	
Rest (ml/min/g)	1.1 ± 0.4
Stress (ml/min/g)	2.2 ± 0.8
Reserve	2.1 ± 0.8

Since CMVD often coexists with CAD, we included all patients with adequate MBFR measurements, regardless of their CAD status or the presence of perfusion defects, in examining the risk factors and disease processes that are associated with decreased MBFR (S1 Table). The unadjusted regression model reveals that traditional cardiovascular risk factors such as age, hypertension, diabetes, and hypercholesterolemia, as well as corresponding lab values, are associated with decreased MBFR. Gender was not associated with MBFR, though women had higher resting MBF than men (S1 Fig). Reduced ejection fraction and increased left-ventricular end-diastolic volume by gated acquisition were associated with decreased MBFR. Cardiovascular diseases such as CAD, CHF, stroke, and PAD were also associated with decreased MBFR. Though the diagnosis of hypercholesterolemia was associated with decreased MBFR, higher total cholesterol and LDL levels were associated with higher MBFR. Statin usage may contribute to this discrepancy. Of note, the presence of heart transplant was not associated with decreased MBFR. The risk factors associated with MBFR were used in an adjusted regression model (S2 Table). Traditional cardiovascular risk factors are associated with lower MBFR, as were CHF and PAD. Interestingly, non-Caucasian race was independently associated with higher MBFR in both the adjusted and unadjusted models (S1 and S2 Tables, S1 Fig).

**Fig 1:**
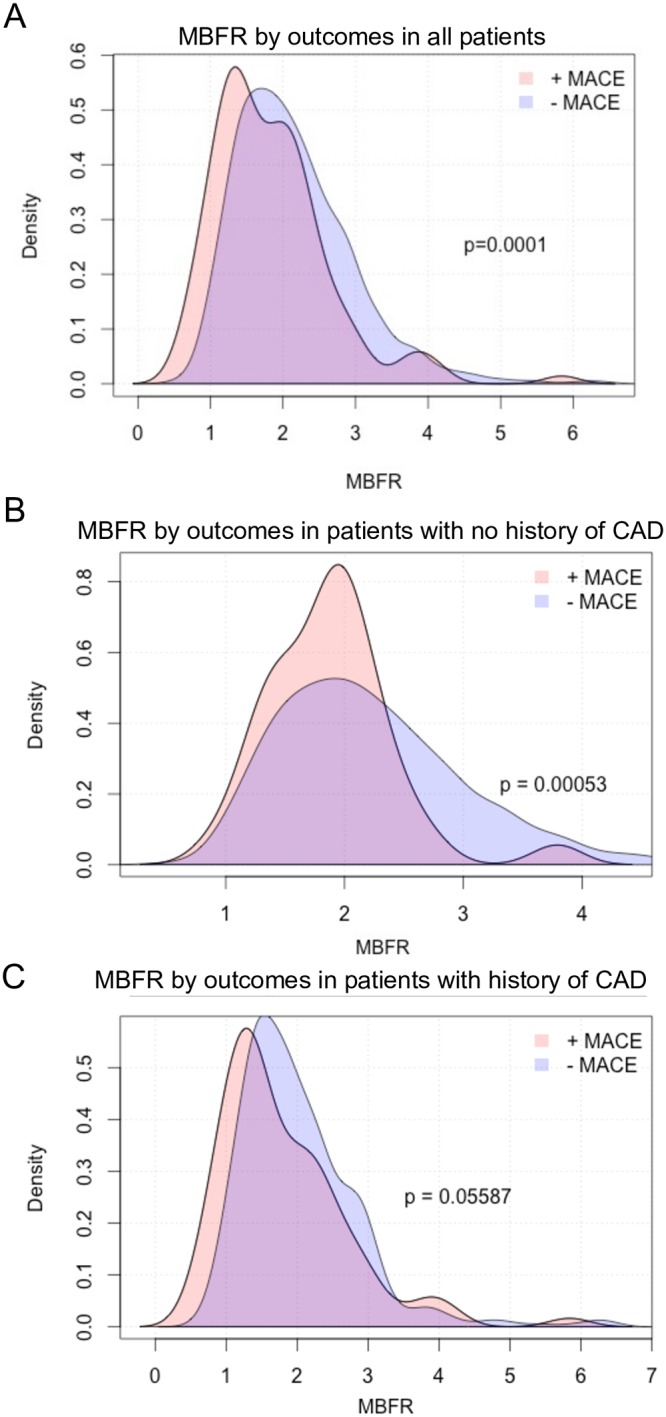
Relationship between MBFR and outcomes. Lower MBFR was associated with increased major adverse cardiovascular outcomes (MACE) (A). This relationship was preserved in patients with no history of CAD by ICD-9 code (B), and there was a trend towards increased MACE in patients with a history of CAD by ICD-9 code (C).

We then examined the relationship between MBFR, cardiovascular risk factors and disease, and outcomes. Decreased MBFR was associated with increased cardiovascular outcomes in an unadjusted analysis (Table 2, Fig 1A, and S3 Fig). Interestingly, increased resting MBF was itself associated with adverse outcomes, though stress MBF was not (Table 2, S2 Fig). Though this result was irrespective of HRBP product, there was an association between resting MBF and hemoglobin (S2 Fig). Since the presence of CAD can confound MBFR measurements, we examined the association between MBFR and outcomes in patients with and without CAD by ICD-9 code. MBFR was associated with outcomes in patients without a history CAD, and we found a similar trend in patients with a history of CAD which was not statistically significant (Fig 1B and 1C).

**Table 2:** Unadjusted regression analysis of cardiovascular outcomes and risk factors.

Risk Factor	Odds Ratio	P-value
PET parameters		
MBFR	0.56±0.08	7.95e-5
Rest MBF	1.98±0.42	1.46e-3
Stress MBF	0.86±0.11	0.21
Perfusion	0.48±0.10	3.54e-4
Demographics		
Age	1.03±0.01	8.80e-5
Gender	1.52±0.29	0.03
Race	0.71±0.11	0.03
Body Mass Index	0.95±0.01	1.06e-6
Smoking Status	1.03±0.10	0.76
Cardiovascular Risk Factors		
Diabetes	1.62±0.31	0.01
Hypercholesterolemia	1.83±0.41	6.38e-3
Obstructive Sleep Apnea	1.09±0.22	0.67
Hypertension	3.49±1.39	1.64e-3
Family History of Cardiac Disease	1.21±0.41	0.57
Chronic Kidney Disease	2.21±0.43	4.31e-5
Renal Transplant	2.31±0.53	2.57e-4
Cardiovascular Diseases		
History of CAD	3.71±0.77	3.22e-10
Congestive Heart Failure	3.12±0.61	5.64e-9
History of Stroke	4.03±0.97	7.58e-9
Peripheral Artery Disease	2.58±0.77	1.44e-3
Heart Transplant	1.24±0.31	0.40

In the adjusted regression model, MBFR was independently and inversely associated with increased cardiovascular outcomes (Table 3, odds ratio 0.72 ± 0.11, p = 0.03). This effect was stronger than that seen with traditional cardiovascular risk factors, such as hypertension and high BMI, but weaker than that of known cardiovascular disease such as CAD, PAD, or stroke. Furthermore, the presence of perfusion defects, which was associated with cardiovascular outcomes in the unadjusted model, was not statistically significant when adjusted for a history of CAD.

**Table 3:** Adjusted regression analysis for outcomes by strength of significant association.

	Odds Ratio	P-value
History of Stroke	2.58±0.68	3.24e-4
History of CAD	2.41±0.57	1.93e-4
Congestive Heart Failure	1.90±0.42	3.95e-3
Renal Transplant	1.83±0.46	0.02
MBFR	0.72±0.11	0.03
Body Mass Index	0.95±0.11	3.48e-5
Hypertension	1.99±0.90	0.13

**Fig 2:**
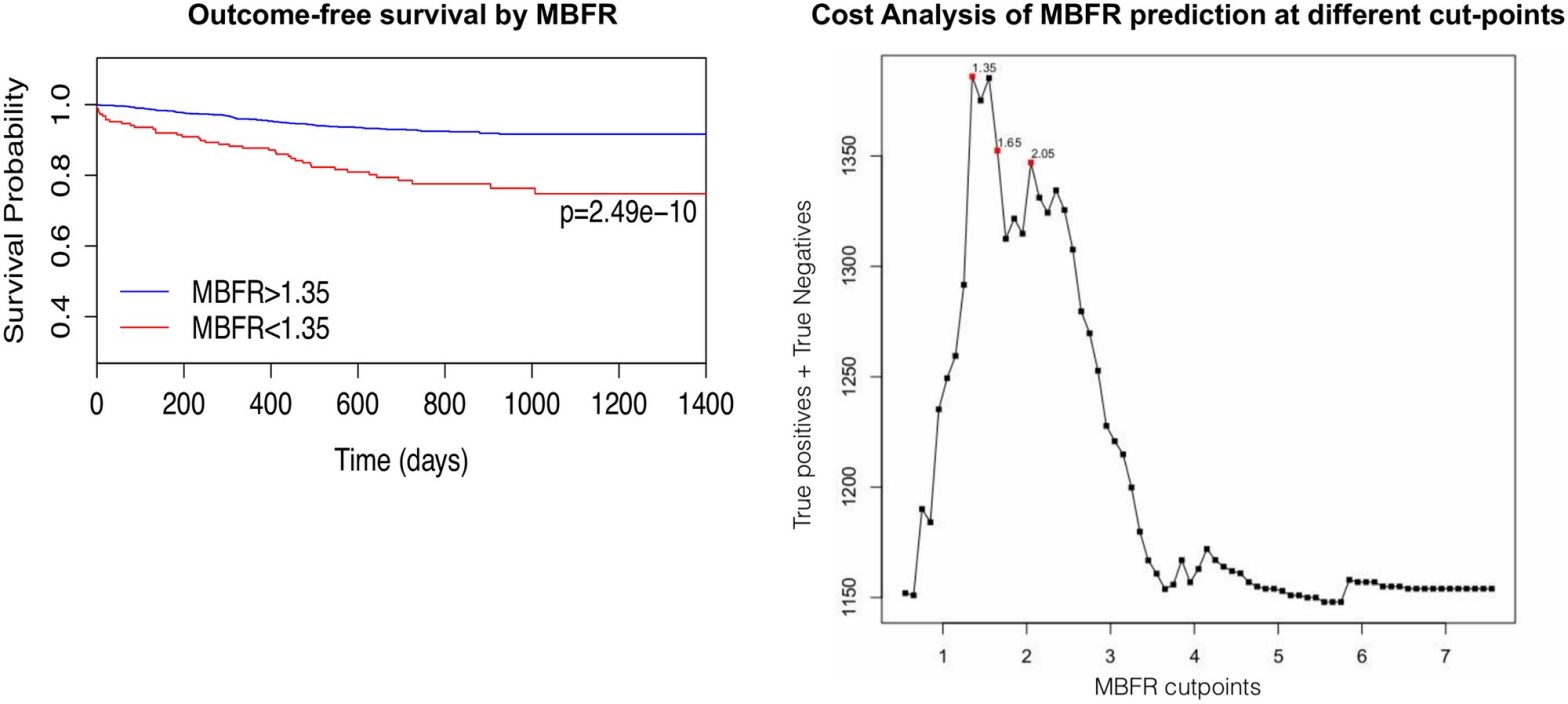
Kaplan-Meier and cost analysis for MBFR thresholds. Kaplan-Meier curve showing decreased freedom from events in patients with MBFR < 1.35 relative to MBFR > 1.35 based on discretization analysis (A). Cost analysis illustrates how the discretization analysis cutpoint maximizes the number of true positives and true negatives (B).

Discretization analysis identified an optimal MBFR cutoff of 1.35 for our cohort of patients which was associated with increased cardiovascular outcomes. Kaplan-Meier curves for MBFR <1.35 and > 1.35 highlight the difference in event-free survival for our cohort (Fig 2A). Cost analysis further illustrates how using discretization analysis identifies an MBFR cut-point that maximizes the number of correct predictions (Fig 2B). MBFR of 2 has been used as a cutoff between normal and abnormal MBFR, and there was a significant difference in outcomes with MBFR of 2 (S3 Fig) [6].

**Fig 3:**
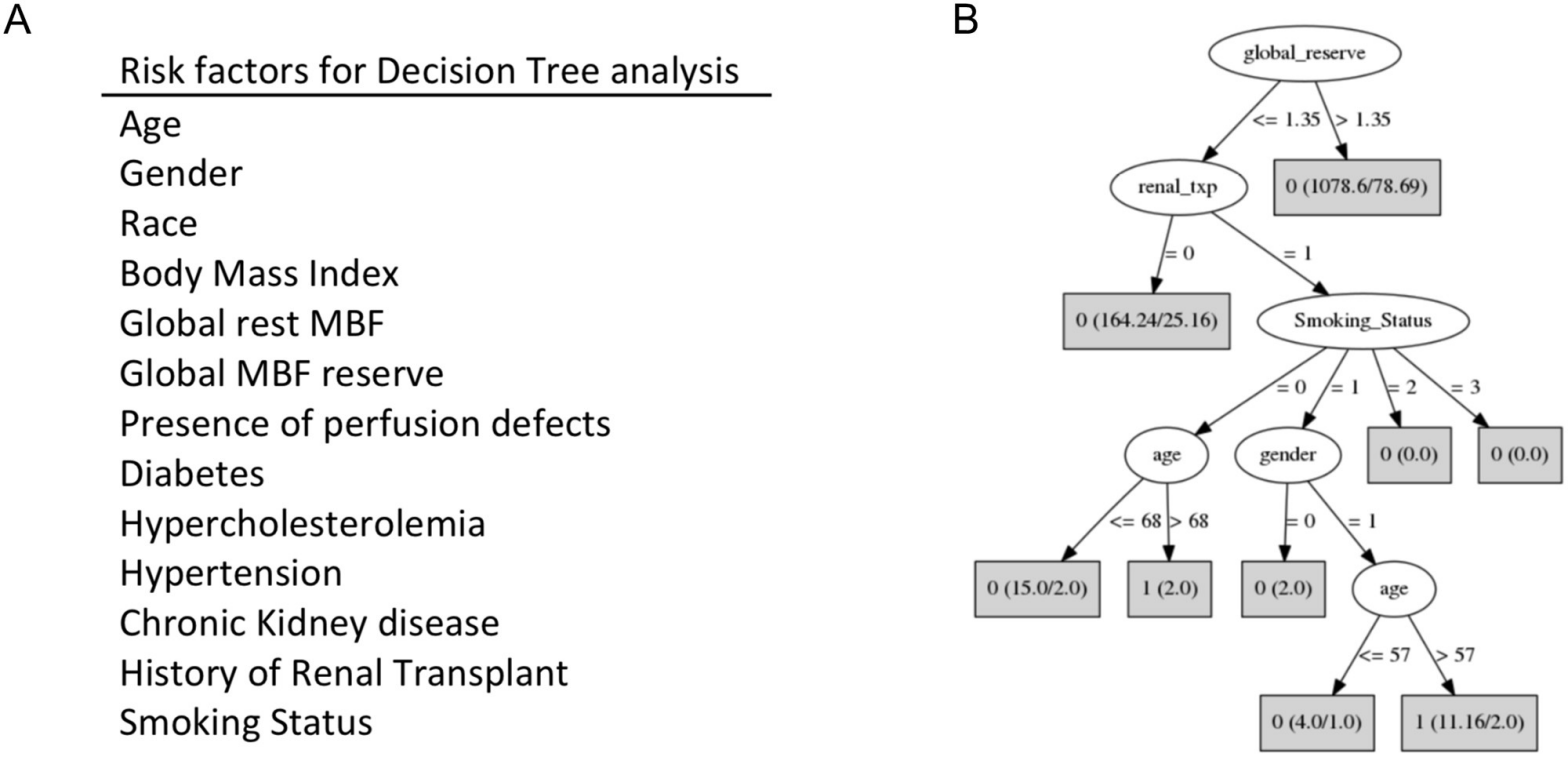
Decision tree analysis. Several risk factors and imaging characteristics were used for the analysis (A). Global reserve was identified in an unbiased way as the first branch off point between outcomes and no outcomes (B). The second most significant factor in outcomes was a history of renal transplant. Rectangles reflect the number of cases that are separated with each branch point node and the number incorrectly classified, if any. Renal transplant = 1 history of renal transplant. Gender = 1 represents male and Gender = 0 represents female. Smoking status is as follows: non-smoker = 0, former smoker = 1, current smoker = 2, unknown smoking status = 3.

Decision tree analysis is a machine learning method which ranks the contribution of risk factors to an outcome. PET imaging parameters, demographics, and risk factors were used for the analysis (Fig 3A). The presence of overt cardiovascular disease such as CAD, CHF, PAD, and stroke were excluded for the analysis since regression analysis showed that they are more strongly associated with outcomes than risk factors. The analysis identified that an MBFR cutoff of 1.35 was the most divisive risk factor between participants that did have an adverse outcome and those that did not in our cohort (Fig 3B). That is, MBFR more accurately differentiated between patients that had cardiovascular outcomes and those that did not compared to all other risk factor. The next branch point was whether a patient had a history of renal transplant. For patients that had low MBFR and had a history of renal transplant, factors such as increased age, male gender, and smoking status were associated with outcomes. Furthermore, other risk factors known to be associated with cardiovascular outcomes, such as hypertension, diabetes, and perfusion defects on myocardial perfusion imaging, did not appear in the decision tree.

## Discussion

We developed a large database of patients (n = 1283) who underwent clinically-indicated cardiac 82Rb PET to examine the association between MBFR, cardiovascular risk factors, and cardiovascular outcomes. MBFR has been difficult to understand and contextualize because of its dichotomous nature; reduced MBFR can represent both a cardiovascular disease, as in ischemia with non-obstructive coronary arteries (INOCA), and a cardiovascular risk factor that modifies prognosis of cardiovascular disease [26].

In the absence of epicardial coronary artery disease, MBFR represents microvascular function and low MBFR represents CMVD. We examined risk factors that affect MBFR and identified hypertension, diabetes, and age as significant risk factors that affect MBFR in an adjusted model. These are known risk factors for CMVD [27]. Additionally, our data showed that African-Americans had increased MBFR relative to Caucasians. There have been similar signals in other studies. For example, African Americans were found to have invasive coronary flow reserve measurements of 4.4 ± 2.3 whereas Caucasian had coronary flow reserve measurements of 4.1 ± 2 [28]. However, this is the first study to robustly show this difference with large number of African American patients using cardiac perfusion PET measurements. The significance of this finding on long-term outcomes in African-Americans will require additional studies. We also found that women had increased resting MBF relative to men, which is consistent with prior reports [29]. Given the association between resting MBF and outcomes, this result warrants future work to understand the pathophysiology of this finding and relevance to long-term outcomes.

In unadjusted analyses for MBFR, CHF was strongly associated with MBFR. Though our study did not differentiate between heart failure with preserved ejection fraction (HFPEF) and heart failure with reduced ejection fraction (HFrEF), there is evidence that microvascular health contributes to the pathogenesis of heart failure [2, 30]. There is likely an interplay between CMVD contributing to heart failure and the disease processes that lead to heart failure causing CMVD. MBFR has also been shown to be correlated to outcomes in patients with heart failure [31]. Further quantification and understanding of microvascular disease in these specific populations will provide additional insight into disease pathogenesis and prognosis.

Depressed MBFR was also found to be independently and strongly associated with adverse cardiovascular outcomes, in agreement with multiple prior studies that have established the association between MBFR and cardiovascular outcomes in various populations [6–10]. In our population, this result was driven predominantly by increased all-cause mortality, which may be a reflection of the high burden of medical comorbidities in this population. The etiology of the link between decreased MBFR and outcomes is still unclear. Though it is possible that that low MBFR increased the risk of death via a direct cardiovascular process, such as myocardial infarction, it is also possible that decreased MBFR serves as a systemic marker of disease, capturing lifetime exposure to systemic risk factors and the association is therefore indirect.

This dichotomy is reflected in the strength of association with outcomes, where MBFR sits between known cardiovascular disease and traditional risk factors. More specifically, in adjusted regression analysis, MBFR was more strongly associated with outcomes than traditional cardiovascular risk factors and less so than having overt atherosclerotic disease. A potential hypothesis for this finding is that MBFR may reflect the cumulative effect of exposure to certain risk factors such as age, diabetes, hypertension, and hypercholesterolemia. MBFR remained independently associated with outcomes after adjusting for history of CAD. Though perfusion defects were associated with outcomes in the unadjusted model, the association disappeared when the analysis adjusted for history of CAD. This suggests that MBFR gives additional information about the risk of outcomes and complements a diagnosis of CAD or the presence of perfusion defects.

Additionally, we found that increased rest MBF was also associated with adverse outcomes. There is evidence that the decreased MBFR often seen in CMVD may be due to increased resting MBF rather than decreased stress MBF [32]. We found this to be true in our data as well, even after correcting for HRBP product. One potential explanation is that patients with increased resting MBF had lower hemoglobin levels, which is itself associated with poor outcomes. A second potential hypothesis is that microvascular disease, which includes luminal obstruction and basement membrane thickening [27], impairs adequate oxygen transport from the vessel lumen to the cardiomyocytes even under basal conditions. The cardiomyocytes may compensate by increasing MBF in hopes of improving oxygen transport, and elevated resting MBF may be evidence of these structural changes.

We next used two unbiased approaches to examine the relationship between MBFR and risk factors. Decision tree analysis determined that MBFR is the most divisive risk factor relative to other cardiovascular risk factors in separating patients who will and will not have outcomes. Furthermore, other risk factors known to be associated with outcomes did not appear in the decision tree. This suggests that traditional cardiovascular risk factors are not as strongly associated with outcomes as MBFR and their effect is captured within the association between MBFR and outcomes.

We also used discretization analysis to identify an MBFR cut-point that best divides patients that did and did not have cardiovascular outcomes. This analysis is independent of the decision tree algorithm and identified the same cutoff of 1.35. Our analysis, as well as others, show that lower MBFR is associated with worsened outcomes regardless of cut-point [33]. Though the discretization analysis is specific to both our patient population and a mean follow-up of 2.3 years, it represents the application of unbiased analysis to partition a continuous variable into two groups. If prospectively validated, this tool could be used by individual institutions to determine appropriate cutoffs to report increased risk of cardiovascular outcomes. It can additionally be used to identify thresholds of MBFR for future studies that would select at risk populations for interventions or therapies. When depressed MBFR is viewed as a disease, determination of a threshold for intervention is important to inform the risk and benefits of directing patient care.

MBF and MBFR were assessed using 82Rb, which is a convenient tracer for clinical use given short half-life and the ability to purchase generator which obviates need of nearby cyclotron. However, it has longer positron range and lower extraction fraction relative to oxygen-15 and N-13 ammonia [34]. Additional limitations include single center and retrospective nature of the study, a referral bias that enriches the study population for cardiovascular disease and therefore cardiovascular outcomes. Future studies will examine the value of MBFR as a prospective predictor of outcomes and determine whether using an MBFR-guided strategy for treatment and cardiovascular risk reduction could affect outcomes. Studies of serial MBFR measurements would also contribute to a better understanding of the progression of CMVD and inform treatment efficacy. For example, statins have been shown to improve MBFR in a short term study [35], but the clinical implications of this improvement, and the benefit of longer term pharmacologic treatment is still unknown. Though obtaining MBFR measurements clinically is currently limited to PET scanners, there is potential to use current SPECT technology to obtain analogous measurements which would further broaden the clinical utility of MBFR [36].

In summary, we used a large and racially diverse clinical population that underwent 82Rb cardiac PET to examine risk factors for decreased MBFR and the association between MBFR and cardiovascular outcomes. We found that rest MBF and MBFR are associated with cardiovascular outcomes. We further stratified MBFR as more strongly associated with outcomes than other established cardiovascular risk factors and less strongly associated with outcomes than established cardiovascular disease. These findings expand the existing literature on MBF and MBFR to a diverse and clinically sicker population and also show that decreased MBFR is more strongly associated with adverse cardiovascular outcomes than traditional risk factors like hypertension, diabetes, and obesity. This suggests that obtaining MBFR measurements may provide additional prognostic information beyond traditional cardiovascular risk factors. Future studies are needed to understand and parse out the contributions of CAD and CMVD to MBFR measurements.

## Supporting information

S1 Fig Gender and racial differences in MBF and MBFR. Women had higher resting MBF than men (A). Caucasian participants had lower MBFR than black participants (B).
(TIF)

S2 Fig The association between rest MBF, outcomes, and hemoglobin. Elevated Rest MBF was associated with increased cardiovascular outcomes (A) and lower levels of hemoglobin (B).
(TIF)

S3 Fig MBFR Kaplan Meier curves. There is a stepwise change in survival between patients with lower MBFR of 1.6 (A) and 2.0 (B). MBFR cutoff of 2 is often used to differentiation between normal and abnormal, and 1.6 is often used to determine significant disease.
(TIF)

S1 Table Unadjusted regression analysis of risk factors and laboratory values associated with MBFR. (PDF)

S2 Table Adjusted regression model of MBFR and risk factors shows the cardiovascular risk factors and cardiovascular diseases that are associated with decreased MBFR. (PDF)

S3 Table Overview of adverse outcomes and association with MBFR. (PDF)

## Abbreviations

CAD: Coronary Artery Disease
CHF: Congestive Heart Failure
CKD: Chronic Kidney Disease
CMVD: Coronary Microvascular Disease
MBF: Myocardial Blood Flow
MBFR: Myocardial Blood Flow Reserve
MPI: Myocardial Perfusion Imaging
PAD: Peripheral Artery Disease
PET: Positron Emission Tomography
