# Supplementary figures and images for: The role of resting myocardial blood flow and myocardial blood flow reserve as a predictor of major adverse cardiovascular outcomes
Source: PLoS One. 2020 Feb 13;15(2):e0228931. doi: 10.1371/journal.pone.0228931 (PMC7018061; doi:10.1371/journal.pone.0228931)

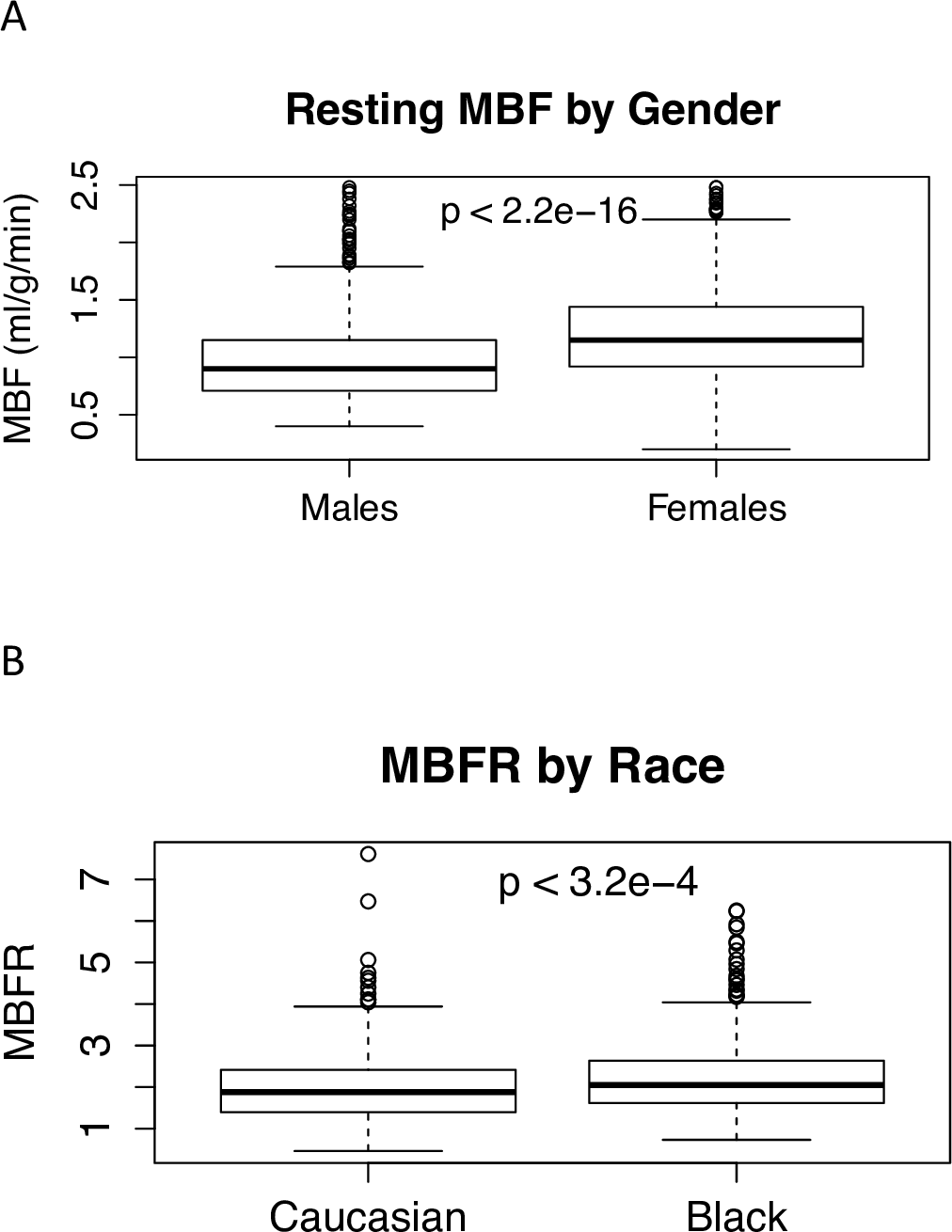

Supplement: S1 Fig — Women had higher resting MBF than men (A). Caucasian participants had lower MBFR than black participants (B). (TIF) [file pone.0228931.s001.tif]

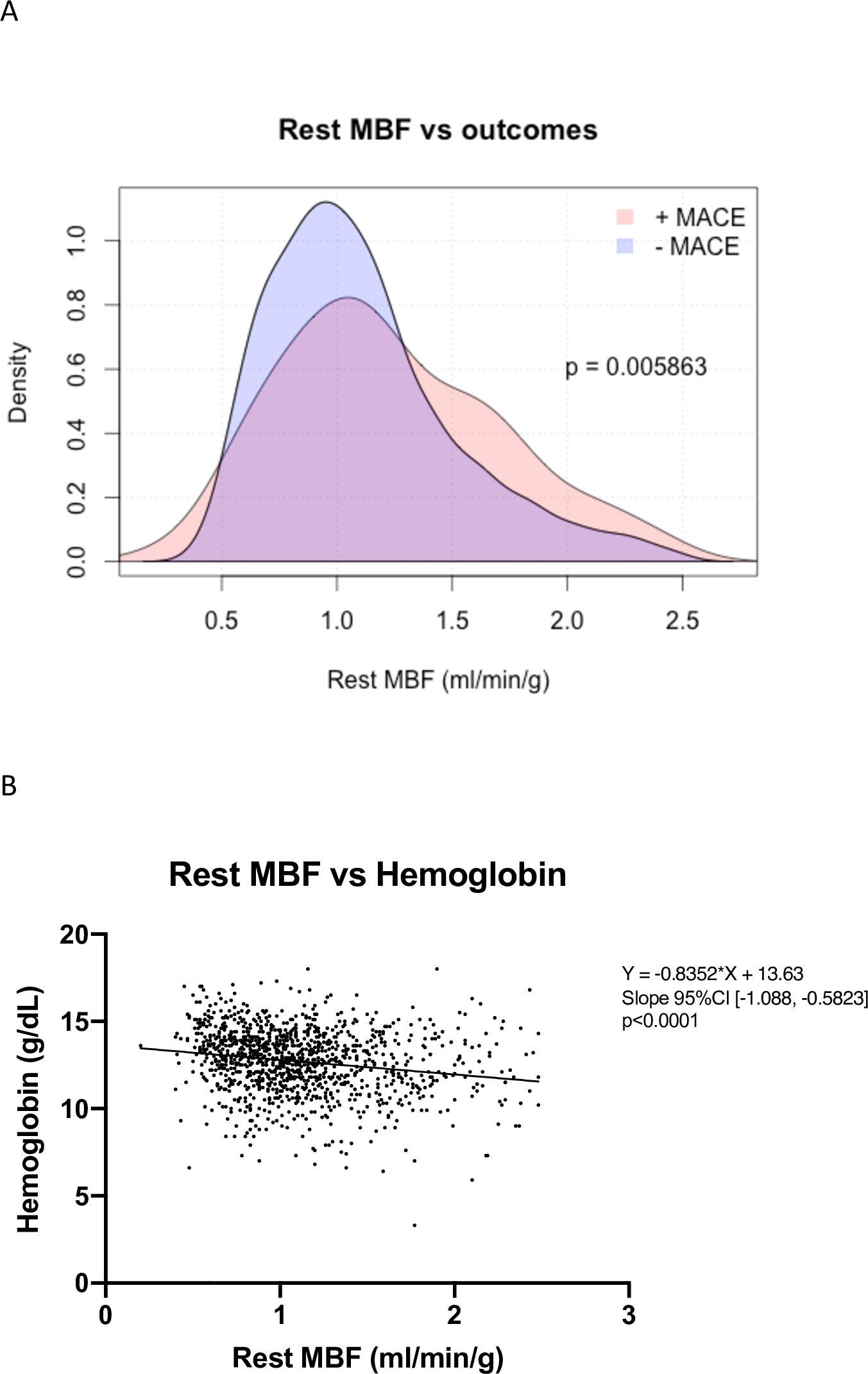

Supplement: S2 Fig — Elevated Rest MBF was associated with increased cardiovascular outcomes (A) and lower levels of hemoglobin (B). (TIF) [file pone.0228931.s002.tif]

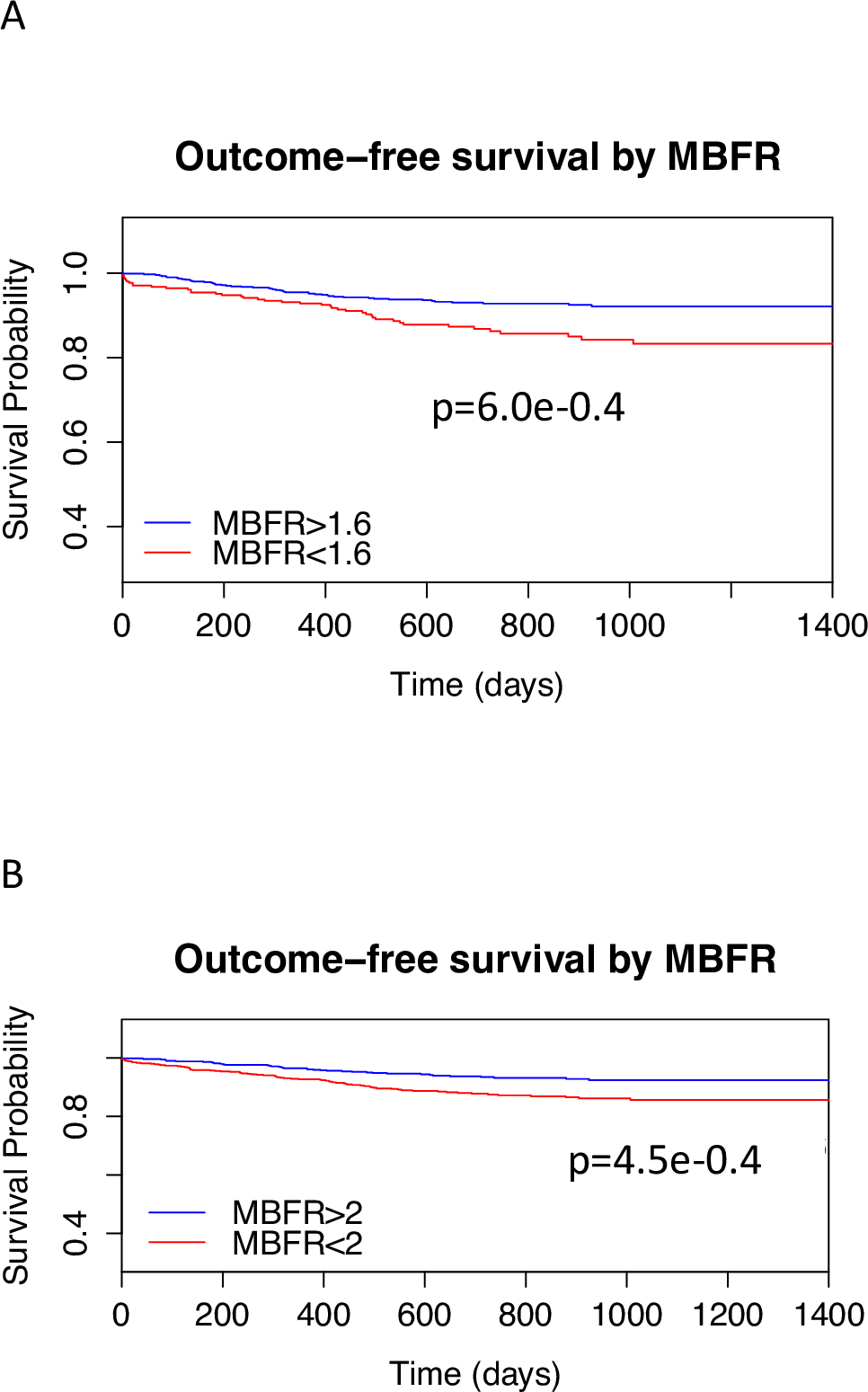

Supplement: S3 Fig — There is a stepwise change in survival between patients with lower MBFR of 1.6 (A) and 2.0 (B). MBFR cutoff of 2 is often used to differentiation between normal and abnormal, and 1.6 is often used to determine significant disease. (TIF) [file pone.0228931.s003.tif]
