# Supplementary material for: The role of resting myocardial blood flow and myocardial blood flow reserve as a predictor of major adverse cardiovascular outcomes
Source: PLoS One. 2020 Feb 13;15(2):e0228931. doi: 10.1371/journal.pone.0228931 (PMC7018061; doi:10.1371/journal.pone.0228931)
Supplement: S1 Table — (PDF) [file pone.0228931.s004.pdf]

S1 Table. Unadjusted regression analysis of risk factors and laboratory values associated with MBFR.

Risk factor	Odds Ratio	P-value
Demographics		
Age	0.988±0.002	8.32e-10
Gender	1.027±0.048	0.57
Race	1.081±0.036	0.02
Body Mass Index	0.999±0.002	0.71
Cardiovascular Risk Factors		
Diabetes	0.785±0.037	4.05e-7
Hypercholesterolemia	0.888±0.043	0.02
Obstructive Sleep Apnea	0.961±0.048	0.42
Hypertension	0.763±0.048	1.74e-5
Family History of Cardiac Disease	0.979±0.085	0.81
Chronic Kidney Disease	0.680±0.032	1.90e-15
Renal Transplant	0.810±0.055	1.83e-3
Smoking Status	1.059±0.038	0.11
Family History of Cardiac Disease	0.979±0.085	0.81
Gated acquisition PET parameters		
Ejection Fraction	1.014±0.002	5.75e-13
End-Diastolic Volume	0.999±0.000	2.00e-3
End-Systolic Volume	0.997±0.001	3.46e-6
Cardiovascular Diseases		
History of CAD	0.801±0.038	3.11e-6
Congestive Heart Failure	0.737±0.038	3.37e-9
History of Stroke	0.900±0.074	0.20
Peripheral Artery Disease	0.640±0.061	2.71e-6
Heart Transplant	0.951±0.062	0.44
Laboratory Values		
Total Cholesterol	1.002±0.001	3.56e-3
HDL	0.999±0.002	0.65
LDL	1.002±0.001	0.02
Triglycerides	1.000±0.000	0.88
Non-HDL	1.000±0.000	0.76
eGFR	0.996±0.001	7.25e-4
Hemoglobin	1.058±0.010	1.19e-8
Hemoglobin A1c	0.985±0.007	0.02
Pro-BNP	1.000±0.000	5.90e-5
