# Supplementary material for: The role of resting myocardial blood flow and myocardial blood flow reserve as a predictor of major adverse cardiovascular outcomes
Source: PLoS One. 2020 Feb 13;15(2):e0228931. doi: 10.1371/journal.pone.0228931 (PMC7018061; doi:10.1371/journal.pone.0228931)
Supplement: S2 Table — (PDF) [file pone.0228931.s005.pdf]

S2 Table. Adjusted regression model of MBFR and risk factors shows the cardiovascular risk factors and cardiovascular diseases that are associated with decreased MBFR.

Risk factor	Odds Ratio	P-value
Age	0.990±0.002	2.31e-7
Race	1.079±0.035	0.02
Diabetes	0.812±0.040	2.88e-5
Hypercholesterolemia	1.113±0.061	0.05
Hypertension	0.863±0.060	0.04
CHF	0.794±0.041	8.50e-6
PAD	0.756±0.071	3.01e-3
