# Supplementary material for: The role of resting myocardial blood flow and myocardial blood flow reserve as a predictor of major adverse cardiovascular outcomes
Source: PLoS One. 2020 Feb 13;15(2):e0228931. doi: 10.1371/journal.pone.0228931 (PMC7018061; doi:10.1371/journal.pone.0228931)
Supplement: S3 Table — (PDF) [file pone.0228931.s006.pdf]

S3 Table. Overview of adverse outcomes and association with MBFR

Outcome	Total #	Odds Ratio	P-value
STEMI	2	1.345+0.933	0.67
NSTEMI	16	1.170+0.320	0.57
UA	13	1.237+0.361	0.47
CVA	22	0.833+0.187	0.42
Death	70	0.220+0.056	2.27E-09
